# Supplementary material for: Adjuvant Immunotherapy After Resected Melanoma: Survival Outcomes, Prognostic Factors and Patterns of Relapse
Source: Cancers (Basel). 2025 Jan 5;17(1):143. doi: 10.3390/cancers17010143 (PMC11719721; doi:10.3390/cancers17010143)
Supplement: Supplementary file 1 [file cancers-17-00143-s001.zip › cancers-3355397-supplementary.pdf]

Adjuvant Immunotherapy After Resected Melanoma: Survival Outcomes, Prognostic Factors and Patterns of Relapse

Sergio Martinez-Recio [1,2,*], Maria Alejandra Molina-Pérez [1], Eva Muñoz-Couselo [3], Alberto R. Sevillano-Tripero [4], Francisco Aya [5], Ana Arance [5], Mayra Orrillo [6], Juan Martin-Liberal [6], Luis Fernandez-Morales [7], Rocio Lesta [8], María Quindós-Varela [8], Maria Nieva [9], Joana Vidal [9], Daniel Martinez-Perez [10], Andrés Barba [1] and Margarita Majem [1,2,*]

- Supplementary Table S1. Distance metastases-free survival (DMFS): median survival time and univariate analysis according to different clinical subgroups……………………….2
- Supplementary Table S2. Overall survival (OS): median survival time and univariate/multivariate analysis according to different clinical subgroups……………...3

Supplementary Table S1. Distance metastases-free survival (DMFS): median survival time and univariate analysis* according to different clinical subgroups.

	mDMFS	HR	P-value**
**Sex**			
-Male	69.5 (26.9-112)	Reference	0.27
-Female	33.3 (13.8-52.8)	1.24 (0.85-1.8)	
**Age** (years old)			0.72
-<65	43.4 (30.4 -56.39)	Reference	
-65-74	69.5 (23.2-115.8)	0.89 (0.57-1.4)	0.61
-≥75	NR (NR-NR)	1.17 (0.65-2.11)	0.61
**Primary tumor type**			**<0.01**
-Cutaneous melanoma	48.8 (31.3-66.3)	Reference	
-Acral melanoma	22.2 (0-49.6)	1.46 (0.78-2.74)	0.24
-Mucosal melanoma	7.4 (4-10.7)	3.95 (1.59-9.82)	**<0.01**
-Unknown origin	26.9 (0-59.9)	1.63 (0.82-3.24)	0.17
**BRAF status**			
-BRAF wild type	48.8 (24.4-73.2)	Reference	0.45
-BRAF mutant	33.4 (25.7-41.1)	1.15 (0.78-1.68)	
**Breslow index** (mm)			0.07
-<0,8	NR (NR-NR)	Reference	
-0,8-2	43.7 (0-NR)	1.2 (0.65-2.67)	0.08
-2-3	27 (8.2-45.8)	1.5 (0.78-2.9)	0.22
-2-4	29 (14.2-43.8)	1.6 (0.79-3.25)	0.19
->4	NR (NR-NR)	0.86 (0.46-1.6)	0.64
**Ulceration**			
**-**Absent	57.3 (22.5-92.1)	Reference	0.67
-Present	43.4 (25.2-61.6)	1.09 (0.72-1.66)	
**LDH**			0.19
-<ULN	33.8 (15-52.6)	Reference	
-ULN - 2xULN	33.3 (13.2-53.4)	1.05 (0.7-1.56)	0.82
-2xULN – 5xULN	NR (NR-NR)	0.56 (0.28-1.1)	0.1
->5xULN	43.6 (26.7-65.7)	0.95 (0.68-1.51)	0.91
**Mitosis** (/mm^2)			0.88
-<1	93.4 (93.4-NR)	Reference	
-1-10	43.4 (22.1-64.7)	1.19 (0.62-2.27)	0.61
**-**≥10	NR (NR-NR)	1.16 (0.58-2.35)	0.67
**Stage** (8th AJCC edition)			0.38
**-**IIB-IIC	NR (NR-NR)	Reference	
**-IIIA-IIIB**	34.2 (12.2-56.2)		
-IIIA	31.7 (11.3-52.1)	6.6 (0.82-52.8)	0.08
-IIIB	34.2 (4.7-63.6)	5.48 (0.74-38.8)	0.1
**-IIIC-IIIID**	55.6 (33.2-77.9)		
-IIIC	49.5 (32.6-66.4)	5.38 (0.74-38.89)	0.1
-IIID	NR (NR-NR)	2.77 (0.29-26.68)	0.38
**-IV**	33.8 (16.78-50.8)	5.3 (0.71-39.62)	0.1
**Time from last resection to start of adjuvant treatment**			
<12 weeks	48.8 (33.2-64.4)	Reference	0.77
>12 weeks	33.2 (9.9-56.5)	1.07 (0.69-1.67)	

*No multivariate analysis was performed since only one variable associated with DMFS in univariate analysis. **P-value <0.05 are marked in bold. AJCC: American Joint Committee on Cancer; CI: confidence interval; HR: hazard ratio; IU: international units; LDH: lactate dehydrogenase; mDMFS: median distant metastases-free survival; mm: millimeters; N: number of patients; NR: not reached; RFS: recurrence free survival; ULN: upper limit of normality.

Supplementary Table S2. Overall survival (OS): median survival time and univariate/multivariate analysis according to different clinical subgroups.

	**mOS** (95% CI)	**Univariate HR** HR (95% CI)	**P-value***	**Multivariate HR** HR (95% CI)
**Sex**				
-Male	NR (NR-NR)	Reference	0.65	
-Female	NR (NR-NR)	0.88 (0.51-1.54)		
**Age**			0.43	
-<65	NR (NR-NR)	Reference		
-65-74	NR (NR-NR)	1.23 (0.67-2.26)	0.51	
-≥75	46.5 (35.1-57.9)	1.64 (0.75-3.57)	0.21	
**Primary tumor type**			**<0.01**	3.9 (1.37-11.11)
-Cutaneous melanoma	NR (NR-NR)	Reference		p=0.01
-Acral melanoma	37.3 (37.3-NR)	2.83 (1.36-5.9)	**<0.01**	
-Mucosal melanoma	28.4 (0-57.8)	3.58 (1.27-10.1)	**0.02**	
-Unknown origin	44.8 (44.8-NR)	2.14 (0.9-5.07)	0.08	
**BRAF status**				
-BRAF wild type	NR (NR-NR)	Reference	0.33	
-BRAF mutant	NR (NR-NR)	0.77 (0.45-1.31)		
**Breslow index** (mm)			0.54	
-<0,8	NR (NR-NR)	Reference		
-0,8-2	NR (NR-NR)	1.81 (0.45-6.25)	0.67	
-2-3	NR (NR-NR)	1.74 (0.59-5.12)	0.31	
-2-4	NR (NR-NR)	1.9 (0.6-6.02)	0.27	
->4	75.7 (65.6-NR)	2.04 (0.78-5.36)	0.15	
**Ulceration**				
**-**Absent	NR (NR-NR)	Reference	**0.04**	1.42 (0.71-2.85)
-Present	NR (NR-NR)	1.96 (1.02-3.77)		p=0.32
**LDH**			0.1	
-<ULN	NR (NR-NR)	Reference		
-ULN - 2xULN	NR (NR-NR)	0.9 (0.5-1.59)	0.73	
-2xULN – 5xULN	NR (NR-NR)	0.33 (0.11-0.96)	0.05	
->5XULN	(NR-NR)	1.71 (0.95-4.98	0.06	
**Mitosis** (mm^2)			0.94	
-<1	NR (NR-NR)	Reference		
-1-10	75.7 (48.8-102.6)	0.9 (0.41-1.98)	0.79	
**-**≥10	NR (NR-NR)	0.96 (0.41-2.34)	0.96	
**Stage**			**0.04**	2.26 (0.92-5.58)
**-**IIB-IIC	NC**			p=0.08
**-IIIA-IIIB**	NR (NR-NR)			
-IIIA	NR (NR-NR)	Reference		
-IIIB	NR (NR-NR)	0.61 (0.11-3.35)	0.57	
**-IIIC-IIIID**	NR (NR-NR)			
-IIIC	75.7 (31.3-120.2)	2.21 (0.53-9.17)	0.28	
-IIID	NR (NR-NR)	2.38 (0.43-13.1)	0.32	
**-IV**	NR (NR-NR)	1.29 (0.27-6.1)	0.75	
**Time from last resection to start of adjuvant treatment**				
<12 weeks	NR	Reference	0.29	
>12 weeks	NR	1.37 (0.76-2.45)		

*P-value <0.05 are marked in bold. **NC: not calculated because of absence of events. AJCC: American Joint Committee on Cancer; CI: confidence interval; HR: hazard ratio; IU: international units; LDH: lactate dehydrogenase; mm: millimeters; mOS: median overall survival; N: number of patients; NC: not calculated; NR: not reached; RFS: recurrence free survival; ULN: upper limit of normality.
